# Allelopathic Effects of Common Landscape and Nursery Mulch Materials on Weed Control

**DOI:** 10.3389/fpls.2018.00733

**Published:** 2018-05-30

**Authors:** Debalina Saha, S. Chris Marble, Brian J. Pearson

**Affiliations:** Department of Environmental Horticulture, Mid-Florida Research and Education Center, Institute of Food and Agricultural Sciences, University of Florida, Apopka, FL, United States

**Keywords:** natural herbicides, secondary metabolites, hardwood chips, pinebark, pinestraw

## Abstract

Use of organic mulch materials such as pinebark, pinestraw, or various hardwood chips for weed control is a common practice in residential and commercial landscapes. Mulch can inhibit weed seed germination and growth through light exclusion, acting as physical barrier, reducing available moisture to weed seeds within the mulch layer, and through release of allelochemicals that may inhibit germination or growth of some weed species. Previous and current research on allelopathic chemicals present in mulch have focused on cover crops and their residues with an emphasis on agronomic crops. These materials would not be suitable in a landscape setting due to rapid decomposition, lack of commercial availability, and little aesthetic appeal. Research is needed concerning identification, quantification, extraction, mechanism of release, persistence, selectivity, genetic regulation, and mode of action of potential allelochemicals present in mulch materials used for landscape purposes. More knowledge of these natural chemicals could aid practitioners and homeowners in the selection of mulch and identify potential new mulch materials that could be utilized in these industries. The purpose of this review is to summarize previous research pertaining to allelopathic compounds present in commonly used mulch materials and identify new potential mulch materials that could be utilized in the landscape sector based upon allelopathic properties. Current areas where additional research is needed are also identified.

## Introduction

The landscape industry represents a diverse network of service companies contributing over $54 billion in sales in the United States (Hodges et al., 2011). Weed management in non-turf areas of residential and commercial landscapes is primarily achieved through application of organic mulch materials that serve as both a weed management tool and provide aesthetic value (Marble, 2015). Materials such as pinebark, pinestraw, hardwood chips from various plant species and other, sometimes inorganic mulches (i.e., gravel or stone) are commonly used due to their low cost and/or availability, and consumer preferences (Chalker-Scott, 2007). Mulch is known to suppress weed growth through light exclusion, by creating a physical barrier, or by reducing available moisture to seeds within mulch layers (Chalker-Scott, 2007). Potential mechanisms of control that have been under-investigated for landscape mulch are allelopathic compounds. An extensive review on landscape mulch materials and their benefits has been published (Chalker-Scott, 2007). Therefore, the focus of this review is to synthesize previous research pertaining to allelopathic compounds present in commonly used landscape mulch materials and to discuss and/or identify new potential mulch materials that could be utilized in the landscape sector based upon allelopathic properties. We also identify current areas where additional research is needed.

## Allelochemicals: Mechanism of Control and Use as Natural Herbicides

Research has shown that mulch primarily inhibits weed growth through light exclusion (Wesson and Wareing, 1967; Popay and Roberts, 1970; Fitter and Hay, 1987; Richardson et al., 2008), creation of a physical barrier (Crutchfield et al., 1986; Facelli and Pickett, 1991; Marble, 2015), and reducing available water within mulch layers (Jordan et al., 2010). While physical characteristics and depth of mulch often explain efficacy in regards to weed control (Chalker-Scott, 2007), allelopathic properties present in some mulch materials may also inhibit weed growth in certain instances.

Molisch (1937) first used the term allelopathy defined as any direct or indirect harmful effect by one plant on another through production of chemicals that are released into the environment (Rice, 1984). Allelochemicals are diverse in chemical structure and produced by plants as secondary metabolites (Paiva, 2000; Hadacek, 2002). They are released by root exudation, volatilization, and death and decay of plants, and through leaching from living or decaying residues (Rice, 1984; Anaya, 1999). Toxicity of these allelochemicals are determined by several factors including concentration, flux rate, age and metabolic state of the plant, and prevailing climate and environmental conditions (Kohli et al., 1993; Wardle et al., 1993; Weidenhamer, 1996; Gallet and Pellissier, 1997; Nilsson et al., 1998). Broadly, allelochemicals can be characterized into *terpenoids* and *phenolics* (Singh et al., 2003). Terpenoids from higher plants include volatile monoterpenes, volatile essential oils, and sesquiterpene lactones (Singh et al., 2003). Among these chemicals, volatile monoterpenes and sesquiterpene lactones show a greater degree of biological activity than others (Singh et al., 2003). Phenolic compounds are hydroxylated aromatic compounds, also referred as “tannins.” Terpenoids and phenolics have been previously investigated in common mulch materials.

Allelopathic production and quantity varies within plant species, cultivar, age, plant organ, and time of the year (Argandona et al., 1981; Hanson et al., 1981; Wyman-Simpson et al., 1991; Devi et al., 1997; Burgos et al., 1999; Cambier et al., 2000). Allelochemicals produced by some species may be toxic enough to lead to death of others (Bewick et al., 1994). Rietveld et al. (1983) reported decline and death of European black alder trees [*Alnus glutinosa* (L.) Gaertn.] due to black walnut (*Juglans nigra* L.) allelopathy. This resulted from a combination of high walnut biomass causing significant release of juglone to the environment and wet soil that restricted aerobic metabolism by soil microorganisms, allowing juglone to build up to toxic levels.

Allelochemicals can also be used for weed management (**Table 1**) as they act as natural herbicides. Advantages of these natural products over synthetic herbicides include: (1) Natural products exhibit structural diversity and possess complex structures that could be used as an alternative for herbicide discovery; (2) Readily decompose in nature; and (3) Contain a different mode of action compared with synthetic herbicides (Duke et al., 1997, 2000; Dayan et al., 1999), providing alternative action sites for herbicides needed to control weeds in areas where few synthetic products can be used (Marble et al., 2015).

**Table 1:** Examples of allelopathic effect of some common plants on weed suppression (partially adapted from Ferguson et al., 2003).

Plant name	Allelochemicals	Target weed/plant	Reference
*Lantana camara* L. (Lantana)	Phenolic compounds	Lantana roots and shoots incorporated into soil reduced germination and growth of milkweed vine (*Morrenia odorata* Lindl.). Fifty percent of milkweed vine seedlings died within 15 days after germination at 1% (w/w) dried lantana root incorporated into the soil.	Achhireddy and Singh, 1984
*Mangifera indica* L. (Mango)	Caffeic acid, ferulic acid, coumaric acid, benzoic acid, hydrobenzoic and cinnamic acid	Dried mango leaf powder (25% extract) inhibited sprouting of purple nutsedge (*Cyperus rotundus* L.) tubers by 85–95%.	Rokiek et al., 2010
*Secale cereale* L. (Rye) *Schedonorus arundinaceus* (Schreb.) Dumort., nom. cons. (Fescue), and *Triticum aestivum* L. (wheat)	Cyclic hydroxamic acid (rye), phenolic acids (wheat)	Suppression of barnyardgrass, redroot pigweed, large crabgrass, etc.	Chase et al., 1991; Ferguson et al., 2003
*Helianthus tuberosus* L. (Jerusalem artichoke)	Salicylic acid, *p*-hydroxybenzaldehyde, *o*-coumarinic acid and coumarin	Residual effects on weed species. Large crabgrass density is inhibited by about 37 and 66% at 30 and 55 days after incorporation of residues respectively.	Tesio et al., 2012
*Crotalaria juncea* L. (Sunn hemp)	Delta-hydroxynorleucine	Growth inhibition of smooth pigweed (*Amaranthus hybridus* L.) by 51% can occur as sun hemp density increases to 100 plants m^-2^.	Collins et al., 2008
*Cichorium intybus* L. (Chicory)	Phenolics and sesquiterpenoid lactones	Inhibition of barnyardgrass and redroot pigweed.	Nishimura et al., 2000; Ferguson et al., 2003
*Cynanchum* L. (Swallow-worts)	Phenolic compounds	Invasive species in northeastern United States and southeastern Canada; inhibited several weed species such as large crabgrass and milkweed.	Ferguson et al., 2003; Douglass et al., 2011
*Tectona grandis* L. f. (Teakwood)	Phenolic acids such as salicylic acid, *p*-hydroxy benzoic acid, chlorogenic acid, and tannic acid	Leaf extracts inhibited jungle rice and sedge. Leaf extracts were able to exhibit 100% inhibition of jungle rice. Methanol extract exhibited sustained inhibitory action (GI∼56–61%) on jungle rice whereas water extract inhibited sedge germination by 25–45%.	Kole et al., 2011
*Avena sativa* L. (oat)	Phenolic acids, scopoletin	*Brassica kaber* (DC) Wheeler var. *pinnatifida* (DC) Wheeler.	Guenzi and McCalla, 1966; Rice, 1984
*Secale cereale* (rye)	Phenolic acids, benzoxazinones	*Taraxacum officinale* F. H. Wigg. (Common Dandelion), *Cirsium arvense* (L.) Scop. (Canada thistle).	Shilling et al., 1985; Barnes and Putnam, 1987; Nair et al., 1990; Mwaja et al., 1995
*Triticum aestivum* L. (wheat)	Phenolic acids, simple acids	*Lolium multiflorum* Lam. (Annual ryegrass).	Guenzi and McCalla, 1966; Shilling et al., 1985
*Sorghum* spp. (sorghum, sudangrass)	Phenolic acids, dhurrin, sorgoleone, *p*-hydroxy benzaldehyde, *p*-hydroxy benzoic acid	Redroot pigweed	Nicollier et al., 1983; Forney and Foy, 1985; Netzley and Butler, 1986; Weston et al., 1989; Einhellig et al., 1993
*Brassica nigra* (L.) Koch (black mustard)	Allyl isothiocyanate, other water-soluble inhibitors	*Phalaris paradoxa* L.	Muller, 1969; Bell and Muller, 1973
*Fagopyrum esculentum* Moench (buckwheat)	Fatty acids	Redroot pigweed	Tsuzuki et al., 1987
*Trifolium* spp. [clover (red, white)]; *Melilotus* spp. (sweetclover)	Isoflavonoids, phenolics	-	Rice, 1984
*Pennisetum typhoides* L. (Pearlmillet)	-	*Trianthemea portulacastrum* L. (Horse purslane).	Narwal et al., 1998; Narwal, 2000


Many investigations have been completed or are ongoing evaluating use of cover crops and their residues for weed suppression. Some results are positive showing enhanced weed suppression and thereby reducing needed herbicide applications, and others with mixed results. Cover crop residue provides a weed-suppressive “mulch” effect due in part by providing a physical barrier, but also due to phytotoxins being released from decomposing residues which impacts weed control selectivity (Putnam, 1988; Weston, 1996; Burgos and Talbert, 2000; Nagabhushana et al., 2001). Rye (*Secale cereale* L.) can provide excellent weed control up to 2 months without affecting yield of crops such as cotton (*Gossypium hirsutum* L.) and soybean [*Glycine max* (L.) Merr.]. Rye allelopathy primarily results from presence of phytotoxins (3H)-benzoxazolinone (or BOA) and 2,4-dihydroxy-1,4- (2H)benzoxazine-3-one (or DIBOA) (Barnes and Putnam, 1987). Research by Burgos and Talbert (2000) showed BOA or DIBOA of rye caused inhibition of germination in small to medium-seeded weed species including Palmer amaranth (*Amaranthus palmeri* S. Watson), large crabgrass [*Digitaria sanguilanis* (L.) Scop.], Indian goosegrass [*Eleusine indica* (L.) Gaertn.], lettuce (*Lactuca sativa* L.), tomato (*Lycopersicon esculentum* L.), and prickly fanpetals (*Sida spinosa* L.). Residues of corn (*Zea mays* L.), oats (*Avena sativa* L.), sorghum (*Sorghum bicolor* L.), and wheat (*Triticum aestivum* L.) and soils in which these crops are grown contain phytotoxins such as ferulic and *p*-coumaric acids. These compounds provide a weed suppressive effect when these crops are produced in no-till or reduced tillage systems and residues left on the soil surface as mulch (Guenzi and McCalla, 1966). Black mustard (*Brassica nigra* L.) also contains allelochemicals that can inhibit germination and seedling growth of wild oat (*Avena fatua* L.) (Turk and Tawaha, 2003). Isothiocyanates, an allelochemicals from mustard species, showed high activity against wheat germination and seedling growth (Bialy et al., 1990). The most active compound, 2-phenethyl ITC, completely inhibited wheat germination at 500 ppm (Bialy et al., 1990). Experiments conducted by Ferguson et al. (2003) showed that application of aqueous extracts of brassica (*Brassica napus* L.), sorghum, and sunflower (*Helianthus annuus* L.) on wheat could successfully reduce weed populations.

## Allelochemicals in Common Landscape Mulch Materials

Duryea et al. (1999) compared chemical, allelopathic, and decomposition properties of six common landscape mulch materials including cypress (*Taxodium distichum* [L.] Rich. and *T. distichum* var. *nutans* [Ait.] Sweet), eucalyptus (*Eucalyptus grandis* W. Hill ex Maiden), pinebark {splash pine (*Pinus elliottii* [Engelm.]) and loblolly pine (*P. taeda* [L.])}, pine needle (*Pinus elliottii* [Engelm.]), melaleuca (*Melaleuca quinquenervia* [Cav.] S. T. Blake), and a utility-trimming mulch (GRU) composed of multiple species [oaks, *Quercus laurifolia* Michx. and *Q. rubra* (L.), *Q. virginiana* Mill., and cherry (*Prunus serotina* Ehrh.)], with a small amount of southern redcedar [*Juniperus virginiana* L. var. *silicicola* (Small) Silba] and southern pines (*Pinus* spp.). Bioassays were conducted by extracting water-soluble chemicals from the mulches followed by application to germinating lettuce seeds and germinants for each mulch extract were recorded. Results showed hydroxylated aromatic compounds were highest in GRU and lowest in melaleuca, pinebark, and pinestraw, but all showed levels of significant activity in bioassay (Duryea et al., 1999). The authors hypothesized allelopathic properties of these mulches could potentially reduce germination of landscape weed species, but were not evaluated.

Rathinasabapathi et al. (2005) demonstrated that water eluates from wood chips of southern redcedar, red maple (*Acer rubrum* L.), swamp chestnut oak (*Quercus michauxii* Nutt.), neem (*Azadirachta indica* A. Juss.), and magnolia (*Magnolia grandiflora* L.) inhibited radicle growth in germinating lettuce. It was also observed that eluates from wood chip mulch of neem, swamp chestnut oak, and red cedar inhibited the hypocotyl growth. Hardwood chips from eucalyptus and cypress, both common in southeast United States, contain more phenolic compounds (tannins) than pinebark and pinestraw (Duryea et al., 1999) and may inhibit germination of weed seeds and seedling growth. Some of the phenolic compounds identified in eucalyptus hardwood are quinic acid, gallic acid, protocatechuic acid, catechin, and chlorogenic acid (Santos et al., 2013).

Maimoona et al. (2011) demonstrated that bark of chir pine (*Pinus roxburghii* Sarg.) and Bhutan pine (*Pinus wallichiana* A. B. Jacks) contained catechin and gallocatechin derivative, quercetin, kaempferol, secoisolariresinol, 3, 4-dihydroxybenzoic acid, and rhamnetin. Previous studies have showed that allelopathy in conifers is due to the presence of phenolic compounds. The above-mentioned phenolic compounds present in the pinebark may be responsible for allelopathy. Terpenoids, such as β-pinene, myrcene, camphor, and cineole, a group of naturally occurring chemicals, have allelopathic effects as toxic, inhibitory or deterrent compounds (Langenheim, 1994). Harman-Ware et al. (2016) reported presence of monoterpenes, α- and β-pinene, camphene, and δ-carene in the terpenoids extract of loblolly pine saplings and pine lighter wood. The β-pinene and camphene are two important potential allelopathic compounds that may be present in pinebark mulch materials and responsible for inhibition of weed seed germination and growth. Further studies are needed to identify the specific allelochemicals present in pinebark mulch, as not much information is available.

Research has been conducted to identify allelochemicals present in pines, and various phenolic acids and their related compounds were isolated from pinebark, needles, and even from soils in pine communities (Lee and Monsi, 1963; Chu-Cho, 1978; Lodhi and Killingbeck, 1982; Kil and Yim, 1983; Son et al., 1996; Node et al., 2003). Kato-Noguchi et al. (2009) identified 9α, 13β-epidioxyabeit-8 (14)en-18-oic acid in the extracts of red pine (*Pinus densiflora*) needles that inhibited root growth of cress (*Lepidium sativum* L.), lettuce, alfalfa (*Medicago sativa* L.), ryegrass [*Lolium multiflorum* (Lam) Husnot], timothy-grass (*Phleum pratense* L.), large crabgrass and barnyardgrass [*Echinochloa crus-galli* (L.) Beauv.] by 9 to 18% and shoot growth of these species by 20 to 65%. It was also observed that with the increase in the extract concentration, there was increased inhibition, suggesting allelopathic potential of red pine needles.

## Potential Mulch Species

Woody plant vegetation management is needed from municipalities and utility companies in order to maintain highway right-of-way visibility and safety, and provide access to utility lines and transformers. This routine maintenance results in large quantities of woody plant debris that is often ground into “utility mulch” and sold for disposal and to recoup costs (Duryea, 2000). Utility mulch varies widely in composition, but often contains a mixture of hardwood species and in many areas such as Florida, a high proportion could contain invasive tree species due to their prevalence. Many invasive plants have been shown to contain high amounts of allelopathic compounds as a defense mechanism, increasing their chance of survival and proliferation (Orr et al., 2005). Several of these species could serve as potential mulch materials due to both their allelopathic properties and frequency in which they are removed following management activities (**Table 2**).

**Table 2:** Allelopathic properties of common and potential mulch species.

Plant name	Allelochemicals	Target weed/plant	Reference
*Ailanthus altissima* (Mill.) (Tree of heaven)	Ailanthone, isolated from this plant has been reported to possess non-selective post-emergence herbicidal activity similar to glyphosate and paraquat	Redroot pigweed, garden cress, foxtail and barnyard grass	Heisey, 1990
*Melaleuca armillaris* (Sd. Ex Gaertn.) (Melaleuca)	1,8-Cineole and terpinen-4-ol essential oils	Radish, garden cress, and canary grass	Farag et al., 2004; Amri et al., 2012
*M. styphelioides* (Sm.) (Melaleuca)	Caryophyllene oxide and spathulenol essential oils	Radish, garden cress, and canary grass	Farag et al., 2004; Amri et al., 2012
*Triadica sebifera* [(L.) Small] (Chinese tallow)	Tannins, phenols, and alkaloids	Loblolly pine	Gresham, 1986
*Schinus terebinthifolia* (Raddi) (Brazilian pepper)	Sesquiterpenes	Lettuce and cucumber	Barbosa et al., 2007
*Juglans nigra* (L.) (Black walnut)	Juglone	Crimson clover, crown vetch, and hairy vetch	Rietveld, 1983
*Pinus halepensis* (L.) (Aleppo pine)	Phenolic compounds present in pine needle/pinestraw	Inhibited growth of tall fescue (*Festuca arundinacea* Schreb.) and bermudagrass (*Cynodon dactylon* [L.] Pers.)	Nektarios et al., 2005
*Eucalyptus grandis* (W. Hill ex Maiden) (Eucalyptus)	Phenolic compounds (tannins) present in hardwood chips	Inhibited germination of lettuce seeds	Duryea et al., 1999
*Pinus elliottii* (Engelm.) (Splash pine)	Hydroxylated aromatic compounds present in pinebark	Inhibited germination of lettuce seeds	Duryea et al., 1999
*Quercus laurifolia* (Michx.), *Q. rubra* (L.), *Q. virginiana* (Mill) (Oaks) (Utility-trimming mulch)	Presence of high amount of hydroxylated aromatic compounds	Inhibited germination of lettuce seeds	Duryea et al., 1999
*Prunus serotina* (Ehrh.) (Cherry) (Utility-trimming mulch)	Presence of high amount of hydroxylated aromatic compounds	Inhibited germination of lettuce seeds	Duryea et al., 1999


Rice (1984) suggested that certain species of melaleuca may have allelopathic properties similar to eucalyptus as both belong to Myrtaceae. Yatagai et al. (1998) studied germination and growth-inhibition activity of five *Melaleuca* species including, *M. saligna* Schau., *M. acacoides* F. Muell., *M. dealbata* S. T. Blake., *M. symphyocarpa* F. Muell., *M. argentea* W. V. Fitzg, *M. bracteata* F. Muell. on radish (*Raphanus sativus* L.) seeds and found that 0.1% of *M. bracteata* leaf oil was able to inhibit the growth and germination of radish seed completely whereas leaf oil from the other five species inhibited growth by 30 to 85%. However, when weed species were tested results were not as promising. Essential oils of *M. armillaris* (Sd. Ex Gaertn.) Sm., *M. styphelioides* Sm., and *M. acuminata* F. Muell. reduced radical elongation of radish, garden cress (*Lepidium sativum* L.), charlock mustard (*Sinapis arvensis* L.), durum wheat (*Triticum durum* L.), and canary grass (*Phalaris canariensis* L.) but did not reduce germination (Amri et al., 2012).

Chinese tallow [*Triadica sebifera* (L.) Small] is an exotic tree that has become highly invasive (Jubinsky and Anderson, 1996). Tallow trees are reported to have allelopathic properties in their leaves, which can alter soil chemistry and can affect native vegetation, negatively (Flack and Furlow, 1996). Tallow has been suspected of allelopathic interference on loblolly pine regeneration as understory vegetation in a multilayered plant community (Gresham, 1986). Previous studies have detected potential allelochemicals such as tannins in tallow leaves and bark (Cameron and LaPoint, 1978; Yang and Kinghorn, 1985) and its leaves are toxic to herbivores (Russell et al., 1969). Chemicals including 6, 7, 8-trimethoxycoumarin and scopoletin, both chemicals belonging to the coumarin group and produced for plant defensive purposes, have been obtained from Chinese tallow bark a root extracts (Yang and Kinghorn, 1985). However, more research is needed to determine what activity, if any, these extracts may have on common weed species.

Brazilian pepper (*Schinus terebinthifolia* Raddi) is another exotic invasive plant in Florida (Morgan and Overholt, 2004). Donnelly et al. (2008) reported reductions in growth and biomass in black mangrove [*Avicennia germinans* (L.) L.] when exposed to the highest density of intact Brazilian pepper fruits growing in 30 ppt (parts salt per 1000 parts seawater) saltwater. They also observed that crushed fruits significantly decreased growth and leaf production of red mangrove (*Rhizophora mangle* L.). The oil content of both leaves and unripe fruit of Brazilian pepper contain sesquiterpenes (from 78.0 to 90.4%) (Barbosa et al., 2007). Sesquiterpenes found in Brazilian pepper have been shown to inhibit radicle growth of lettuce (88.6–92.4%) and cucumber (*Cucumis sativus* L.) (50.5–84.5%) at 10,000 μg mL^-1^ concentration (Barbosa et al., 2007).

Tree of heaven [*Ailanthus altissima* (Mill.) Swingle] is another potential plant species for landscape mulch production. The extracts from the fresh leaves of tree of heaven showed germination/growth inhibitory effect against alfalfa in laboratory bioassays (Tsao et al., 2002). De Feo et al. (2003) reported that aqueous root extract of tree of heaven exhibits allelopathic activity against radish, garden cress, and common purslane (*Portulaca oleracea* L.) seeds, the latter two species being important weed species. Heisey (1990) tested the allelopathic effect of root bark extract of tree of heaven on seven herbaceous plant species including redroot pigweed (*Amaranthus retroflexus* L.), garden cress, velvetleaf (*Abutilon theophrasti* Medik.), foxtail [*Setaria pumila* (Poir.) Roem. and Schult.], barnyard grass, garden pea (*Pisum sativum* cv. ‘Sugar Snap’), and corn (*Zea mays* L.) by applying to seeds pre- and post-emergence. Root bark extract showed a strong herbicidal activity when applied to greenhouse soil both pre- and post-emergence to all seven species. Post-emergence application resulted in complete mortality of all species with the exception of velvetleaf.

Black walnut trees are native to the United States and often found growing on landscape sites with other shade trees. They produce a non-toxic, colorless chemical known as hydrojuglone present in leaves, stems, fruit hulls, inner bark and roots. When hydrojuglone is exposed to soil compounds or air, it is oxidized into allelochemicals juglone, which is extremely toxic (Appleton et al., 2000). Rietveld (1983) determined juglone sensitivity of 16 species including {crimson clover (*Trifolium incarnatum* L.), crown vetch (*Coronilla varia* L.), hairy vetch (*Vicia villosa* Roth.), Korean lespedeza (*Lespedeza stipulacea* Maxim.), sericea lespedeza [*L. cuneata* (Dumont) G. Don], ginnala maple (*Acer ginnala* Maxim.), Siberian peashrub (*Caragana arbor-escens* Lam.), Russian olive (*Elaegnus angustifolia* L.), autumn olive (*E. umbellata* Thunb.), amur honeysuckle (*Lonicera maackii* Maxim.), white oak (*Quercus alba* L.), white ash (*Fraxinus americana* L.), yellow poplar (*Liriodendron tulipifera* L.), European black alder, eastern white pine (*Pinus strobus* L.), and Scotch pine (*P. sylvestris* L.)}. He reported that amur honeysuckle, sericea lespedeza, crimson clover, European black alder, and autumn olive were most sensitive to juglone in terms of seedling shoot elongation and dry weight accumulation at concentration as low as 10^-6^ M. Whereas, all other species were wilted and eventually died by 10^-3^ M juglone, and most of them were chlorotic and extremely stunted at 10^-4^ M juglone. Juglone and decomposed walnut leaf sap can also negatively affect root and stem development of muskmelon (*Cucumis melo* L.) and cucumber seedlings and the negative effect deceases as dilution ratios of decomposed leaf juice increases (Terzi, 2008). While black walnut is a high value species for timber (Campbell and Dawson, 1989), residual materials that are lost during harvesting procedures could potentially be used in certain landscape situations.

## Knowledge Gaps and Future Prospects for Research

Although research has focused on allelopathic properties of various agronomic crop residues and cover crops, and their effect on weed suppression or potential as natural herbicides/herbicide templates (Weston, 2005), these materials would not be suitable in landscapes due to rapid decomposition, availability, and appearance (Marble, 2015). There remains a significant knowledge gap concerning identification and quantification of potential allelochemicals present in the common landscape mulch materials. In addition to characterizing allelopathic properties of current and potential mulch materials (**Table 2**), further investigation of the mechanism of action of allelochemicals is also deserved (Weston, 2005). Another aspect to consider is the extraction process of the newly identified allelochemicals from landscape mulches and if their persistence in the environment in sufficient concentration to affect weed species (Ferguson et al., 2003). From a commercial perspective, further work is also needed to identify how aging and handling these materials after harvest impacts allelochemical composition, as age has been shown to have a significant effect (Achakzai et al., 2009). Mulch availability typically varies by region and what materials are available locally (Marble, 2015). A better understanding of potential allelopathic effects of these mulch materials could be used by mulch manufacturers for promotion and to aid the horticulture industry in selecting mulch for different applications. Due to a high degree of variability in both allelopathic potential and weed species response reported here, it is important that researchers identify key characteristics of mulch materials used including plant species, age, plant parts used, and harvesting and handling procedures prior to experimentation. Identifying activity of these compounds on economically important weed species, in lieu of bioassay species such as lettuce or radish, would also be beneficial from a weed management perspective.

## Author Contributions

DS and SM reviewed and wrote the manuscript. DS, SM, and BP gathered information from various sources.

## Conflict of Interest Statement

The authors declare that the research was conducted in the absence of any commercial or financial relationships that could be construed as a potential conflict of interest.
